# Polymicrobial infection in cystic fibrosis and future perspectives for improving *Mycobacterium abscessus* drug discovery

**DOI:** 10.1038/s44259-024-00060-5

**Published:** 2024-11-14

**Authors:** Emily, J. Baker, Gemma Allcott, Jonathan A. G. Cox

**Affiliations:** College of Health and Life Sciences, Aston University, Aston Triangle, Birmingham, B4 7ET UK

**Keywords:** Clinical microbiology, Antibiotics

## Abstract

Polymicrobial communities inhabit the cystic fibrosis (CF) airway, whereby microbial interactions can occur. One prominent CF pathogen is *Mycobacterium abscessus*, whose treatment is largely unsuccessful. This creates a need to discover novel antimicrobial agents to treat *M. abscessus*, however the methods used within antibiotic discovery are typically monomicrobial. This review will discuss this pathogen whilst considering the CF polymicrobial environment, to highlight future perspectives to improve *M. abscessus* drug discovery.

## Introduction

### Reshaping the monomicrobial perspective

Throughout history, our understanding of medical microbiology has held a monomicrobial bias, influenced by the publication of ‘Koch’s postulate’ by Robert Koch in 1890^1^. This postulate formed the criterion for establishing a causal relationship between a microbe and a disease, including the idea that a pathogen will only cause an infectious disease if it can be isolated from all diseased individuals. Despite setting the foundation for knowledge in microbiology, Koch’s postulate has been criticized for multiple reasons, including the monomicrobial perspective it infers by suggesting that only one microbe will be associated with one disease^1^. Numerous diseases instead demonstrate a polymicrobial aetiology, including cystic fibrosis (CF)^2^, whereby microbial ecosystems can develop and influence a therapeutic outcome^3–5^. It is therefore timely to explore infection from a polymicrobial perspective, as would be exhibited in vivo^1^. In this review, we highlight the importance of reshaping this monomicrobial perspective, including a summary of the current knowledge surrounding the polymicrobial nature of CF. A specific focus will be on how application of this polymicrobial environment could increase the physiological relevance of *Mycobacterium abscessus* drug screening and discovery, with this species being less studied compared to its CF-associated counterparts.

### An introduction to *M. abscessus*—an emerging opportunistic pathogen

*M. abscessus* was first recognised in the 1950’s from a patient’s knee abscess, being described as an ‘unusual’ acid-fast bacterium that phenotypically differed to that of the pre-established tubercule bacilli. Being more rapidly growing and dissimilar in its biochemical properties compared to *Mycobacterium tuberculosis*, the isolate was proposed as the new species *Mycobacterium abscessus*, owing to the location of its initial isolation^6^. Such discrepancies therefore placed *M. abscessus* as a type of non-tuberculous mycobacteria (NTM), a group of pathogens consisting of around 200 genetically distinct species that are all outside of the tuberculous realm^7^.

**Fig. 1:**
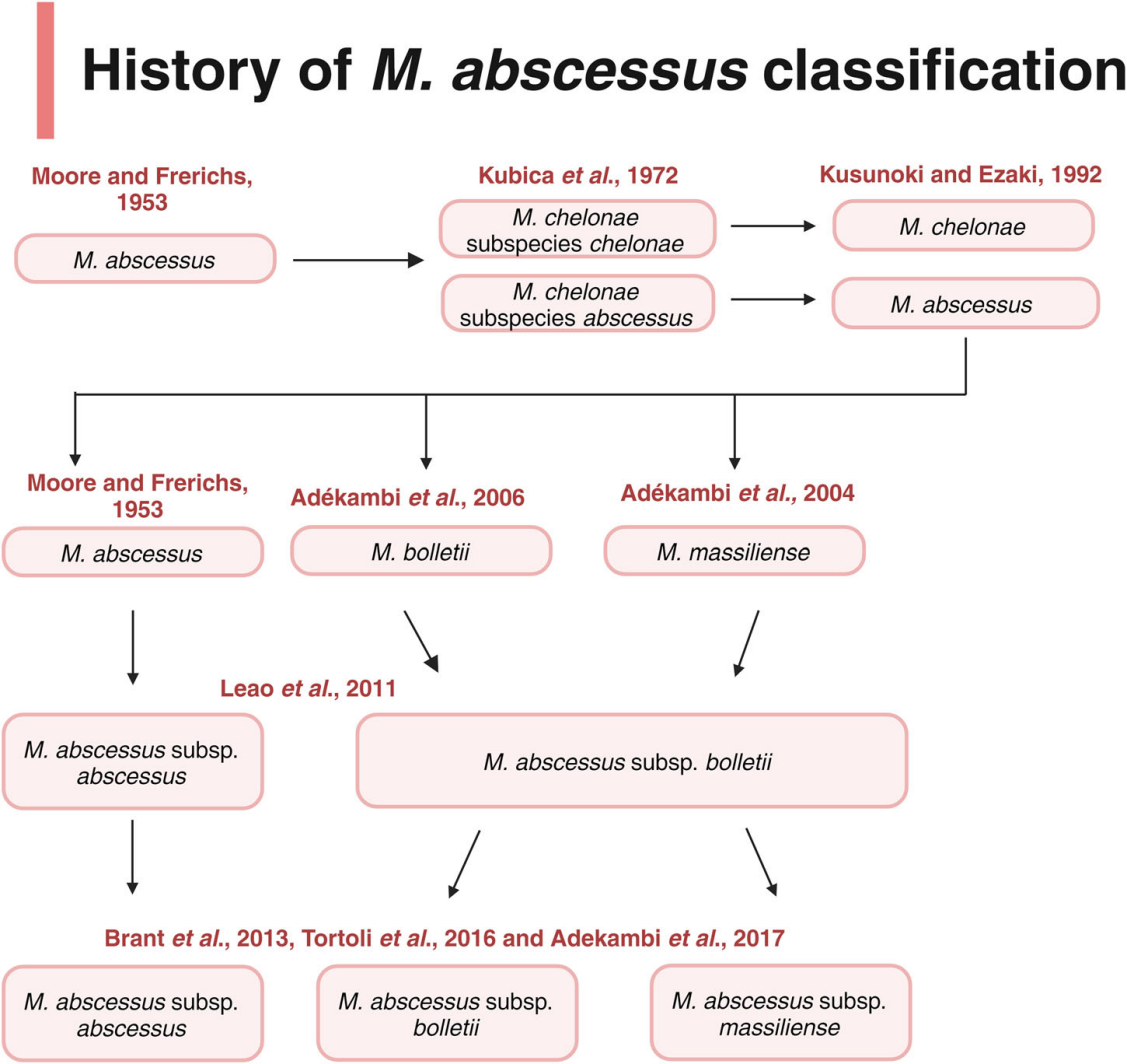
Diagrammatic summary of the history of MABSC classification. Since its first isolation in 1953, the genomics of the species have been scrutinised until three distinct subspecies were later agreed upon^6–13,121^. Figure created on Biorender.com, accessed on 13 April 2023.

Our understanding of *M. abscessus* has progressed significantly since its first isolation, now being accepted as part of a complex of three defined subspecies that are collectively referred to as the *Mycobacterium abscessus* complex (MABSC). Namely, *M. abscessus* subsp. *abscessus*, *M. abscessus* subsp. *bolletii*, and *M. abscessus* subsp. *massiliense*^7–9^ form MABSC, however the taxonomic position of these subspecies has followed assessment over recent years. Initially, *M. abscessus* was believed to be genomically homogeneous with *M. chelonae*, and thus it was denoted a subspecies of this pathogen^10^. In 1992, however, *M. abscessus* was promoted to species status, because of a degree of genomic and phenotypic diversity between *M. chelonae* subsp. *abscessus* and *M. chelonae* subsp*. chelonae*. For example, *M. chelonae* subsp. *chelonae* was shown to utilise citrate as a carbon source, whereas *M. chelonae* subsp. *abscessus* is unable to metabolise citrate^11^. Later identification of *M. massiliense* and *M. bolletii* in 2004 and 2006, respectively, suggested that there are three defined subspecies^12,13^, however their need to be placed as distinct entities remains under debate. Figure 1 diagrammatically represents the categorisation of these three subspecies, from 1953 through to present day.

Like many of the infectious agents associated with CF, MABSC are ubiquitous microbes, preferentially infecting susceptible patient populations including individuals with CF, bronchiectasis and immunosuppression^14,15^. It is now considered one of the most common rapidly growing mycobacteria isolated worldwide^16^, effecting both adult and paediatric patients across the globe^17,18^. Specifically, it is a common NTM isolated in Australia and Sweden, and is the second and third most common NTM in South Korea and Taiwan, respectively^16^. In addition, *M. abscessus* is on the rise in the United Kingdom, with 44% of NTMs isolated in London (England) being identified as rapidly growing mycobacteria in 2008^16^. Despite this global threat, treatment of MABSC remains incredibly challenging, with only a 45.6% cure rate being identified in one study^19^. There is therefore a huge demand to uncover novel therapeutic agents that can successfully treat MABSC with minimal treatment burden. Despite this need, our current in vitro models are poor predictors of in vivo conditions, therefore significantly compromising drug discovery efforts^20,21^. The influence of the complex polymicrobial communities on MABSC remains one of the lesser studied interactions, however, this could offer significant potential to improve the physiological relevance of in vitro drug screening and discovery.

### Antibiotic resistance in *M. abscessus*

MABSC has phenomenal intrinsic resistance to antibiotics^22^. The most widely recognised resistance property within mycobacteria is it’s thick, waxy cell wall, that limits the permeability of antimicrobials into the cell^23^. This structure consists of a plasma membrane, a mycolic acid, arabinogalactan and peptidoglycan complex (MAPc) and a capsule-like polysaccharide material, forming a complex structure unique to the mycobacteria genus^24^. The α-alkyl and β-hydroxy mycolic acids form an outer lipid layer that confers its waxy and hydrophobic nature, thus limiting the permeability to hydrophilic compounds. In addition, MAPc acts to structurally support the robustness of the upper myco-membrane, where the capsule-like layer resides^24,25^. This strength and hydrophobicity contribute to the drug resistance exhibited by Mycobacterial species. Additionally, efflux pumps can reside within this membrane, that can remove antibiotics (such as clarithromycin) from the cell to confer resistance^26^.

Furthermore, inducible macrolide resistance is commonly observed within MABSC, which is one distinguishing factor that supports the subspeciation of MABSC isolates (Section “An introduction to *M. abscessus*—an emerging opportunistic pathogen”)^7^. This is attributed to the presence of erythromycin ribosome methyltransferase 41; a macrolide resistance gene that is typically deleted in *M. abscessus* subsp. *massiliense* despite being present within *M. abscessus* subsp. *bolletii* and *M. abscessus* subsp. *abscessus*^27^. This gene can be activated by the presence of macrolides (such as clarithromycin), thus categorising this as an inducible resistance mechanism^28^. The transcriptional regulator WhiB7 is similarly inducible, being induced in the presence of antibiotics such as tetracycline and amikacin^29^.

**Fig. 2:**
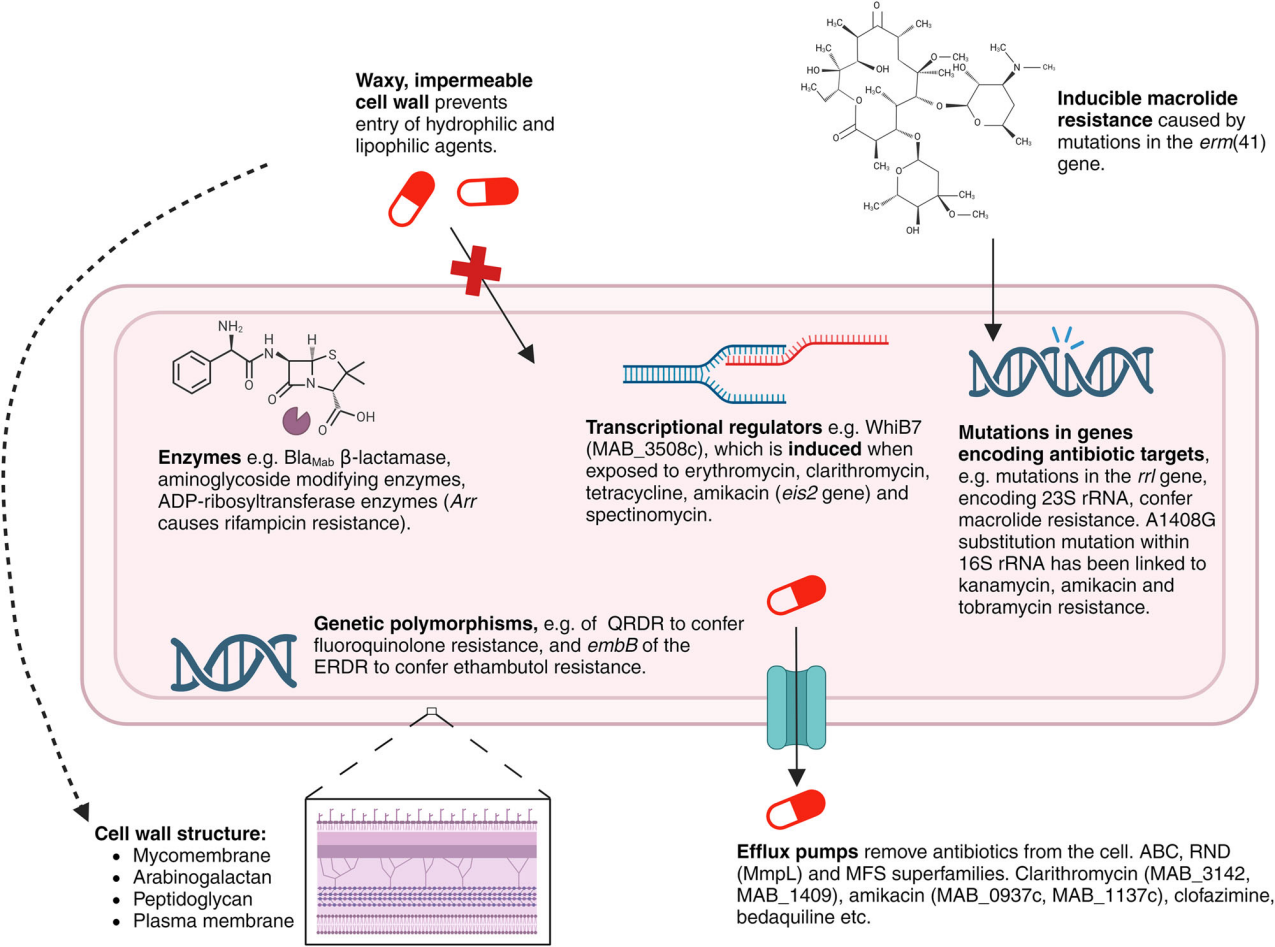
Schematic of the antibiotic resistance mechanisms within *M. abscessus*^23–27,29–32,122–129^. Figure created on Biorender.com, accessed on 21 August 2024. ABC adenosine triphosphate-binding cassette, ADP adenosine diphosphate, Arr ADP-ribosylating rifampicin resistance gene, Bla_Mab_ *Mycobacterium abscessus* β-lactamase, *eis2* enhanced intracellular survival 2, *embB* ethambutol resistance gene B, ERDR erythromycin resistance determining region, *erm*(41) erythromycin ribosome methyltransferase 41, MFS major facilitator superfamily, MmpL Mycobacterial membrane protein Large, RND resistance-nodulation-division, rRNA ribosomal ribonucleic acid, QRDR quinolone resistance determining region.

Another genetically-mediated macrolide mechanism is the presence of *rrl* gene mutations, that, in its functional state, encodes the macrolide target 23S rRNA^30^. Genetic polymorphisms can also induce resistance to a different antibiotic classes, including polymorphisms in the quinolone resistance determining region of Gyrase A that confer fluoroquinolone resistance^31^. Moreover, *M. abscessus* expresses the β-lactamase Bla_Mab_; an enzyme capable of hydrolysing the β-lactam ring to induce β-lactam resistance^32^. Together, these resistance properties make for a very challenging and complex treatment regimen^19^. Figure 2 provides a schematic representation of these resistance properties in more detail.

## Polymicrobial infection in cystic fibrosis

### The CF lung environment facilitates the colonisation of diverse polymicrobial communities

The pathophysiology of the CF airway creates an environment perfectly suited for *M. abscessus* colonisation, however NTMs are not the only pathogens associated with CF. Rather, CF infection is polymicrobial, varying between patients^2^.

CF is characterised by autosomal recessive mutations within the CF conductance regulator (CFTR) gene^33,34^, resulting in defects to the CFTR ion channel^34^. This protein functions as a chloride channel and is also a regulatory protein for the epithelial sodium channel, and as such, defective salt transport occurs across epithelial cells when this protein is defective^34,35^.This dysfunction results in highly viscous sputum, promoting improper mucociliary clearance^36,37^. In its functional state, this has been elucidated as the primary innate defence mechanism in the airways^38^, and thus absence of functional mucociliary clearance compromises pathogen defence to facilitate the habitation of microbes. Complex bacterial communities can develop within CF lung, with common species including *Pseudomonas aeruginosa*, *Staphylococcus aureus*, *Haemophilus influenzae*, NTMs, *Stenotrophomonas maltophila*, *Aspergillus fumigatus* and members of the *Burkholderia cepacia* complex^39,40^. These are not the only species to be associated with CF; one study isolated 1837 different taxa in a cohort of 63 CF patients, highlighting the polymicrobial nature of CF bacterial communities. The species comprising these bacterial communities typically vary depending upon the patient, thus highlighting the widespread diversity that is commonly observed within CF. This is particularly true for younger CF patients, with older subjects showing a progressive loss of bacterial diversity over time^2^.

### Interspecies interactions within CF polymicrobial communities

The complexity associated with polymicrobial communities relates to the diverse interspecies interactions that can take place between microorganisms, which can be categorised as either ‘cooperative’^41^ or ‘antagonistic/competitive’^42^. The most studied interaction is that between *P. aeruginosa* and *S. aureus*, with these species being common CF microorganisms (isolated in 73% and 65% of samples, respectively)^2^. The co-existence of these two species results in changes to a variety of physiological, genetic and biochemical characteristics, including an induction of exploratory motility in *P. aeruginosa*^43^, a switch to the small colony variant phenotype in *S. aureus*^44,45^ and alterations in nutrient utilisation by the two microbes through nutrient exchange^46^.

*P. aeruginosa* has been reported to have a competitive advantage over *S. aureus*, influenced by the two-component system NtrBC—expression of which is induced in *P. aeruginosa* in the presence of *S. aureus*. To investigate this, the activity of the *ntrBC* promoter in *P. aeruginosa* was measured by luminescence detection in the presence and absence of *S. aureus*. At twelve hours post-inoculation, the activity of the *ntrBC* promoter was 5.1-fold greater in co-culture compared to *P. aeruginosa* monoculture. When *ntrBC* was knocked out of *P. aeruginosa*, *S. aureus* could outcompete *P. aeruginosa*—a finding opposite to the competitive advantage shown by the *P. aeruginosa* wildtype. This therefore indicates that the NtrBC system is capable of moderating the competition between these two species^47^.

**Fig. 3:**
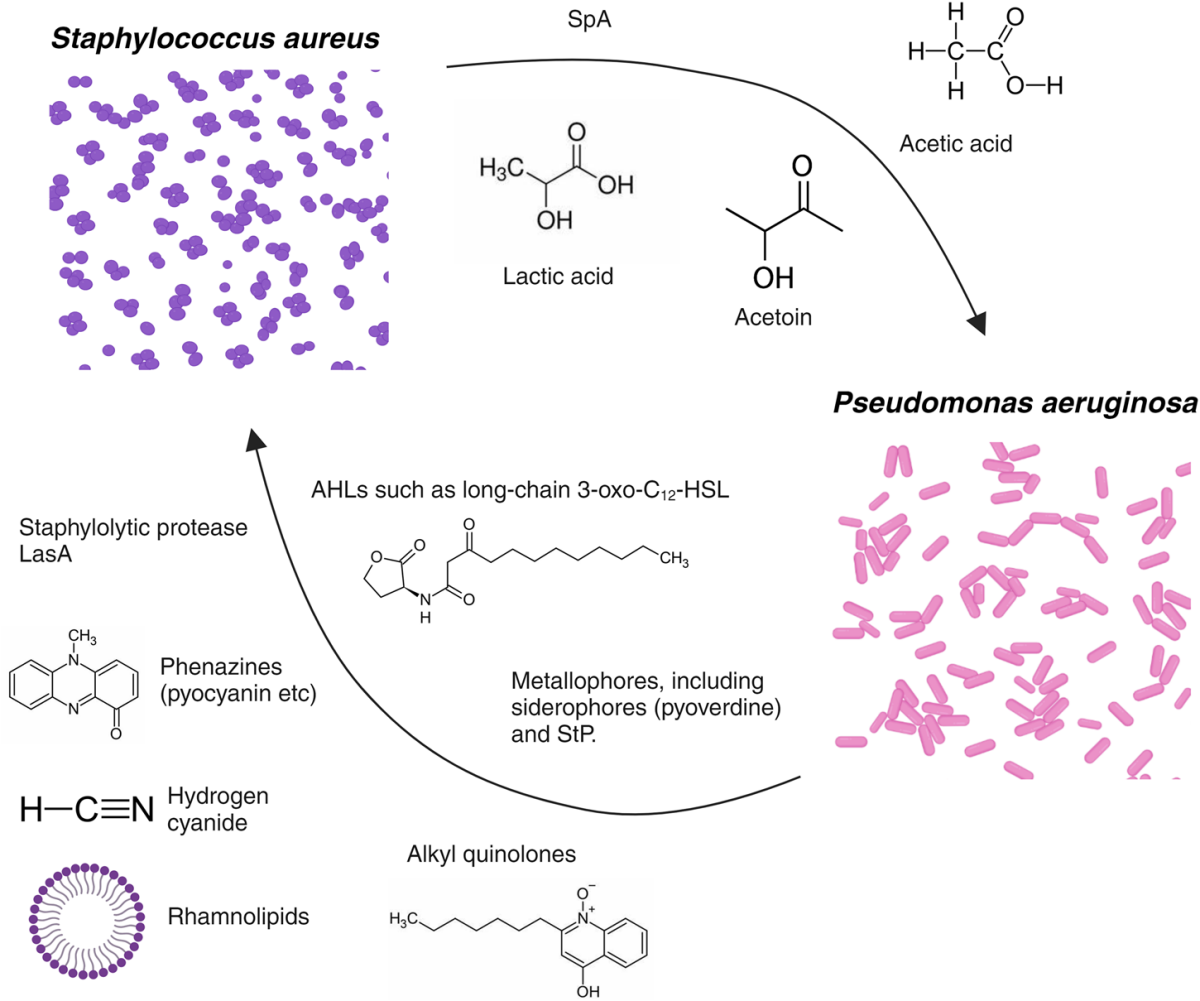
Inhibitory molecules involved in the interplay between *S. aureus* and *P. aeruginosa.* The pseudomonal ‘alkyl quinolones’ encompasses 2-heptyl-3-hydroxy-4(1H)-quinolone (or Pseudomonas quinolone signal); 4-hydroxy-2-heptylquinoline-*N*-oxide (HQNO) and 2-heptyl-4-hydroxyquinoline (HHQ)^47,48,130–136^. Figure created on Biorender.com, accessed on 21 August 2024. AHL N-acylhomoserine lactones, 3-oxo-C12-HSL *N-*(3-Oxododecanoyl)-L-homoserine lactone, SpA *Staphylococcus aureus* protein A, STP staphylopine.

Despite this finding, a mutual antagonism still exists between the two species, which is facilitated by the initial separation of these pathogens in the lung^48^. Throughout the life of a CF patient, the microbial ecosystem within the respiratory tract will undergo continuous changes, with colonisation of *S. aureus* and *H. influenzae* in childhood typically preceding *P. aeruginosa* colonisation later in life^49^. *S. aureus* is thought to establish a bacterial community prior to pseudomonal infection, that will begin secreting *P. aeruginosa*-inhibiting substances in the presence of glucose^48^. Glucose is typically elevated within CF sputum, with a mean concentration of 700 µmol/L^50,51^; therefore suggesting that the composition of CF sputum supports this co-colonisation process. The metabolism of glucose by *S. aureus* creates organic acid products (such as acetic acid), that have been shown to kill *P. aeruginosa*^48,52^. Such mechanism creates a barrier for *P. aeruginosa* infection surrounding *S. aureus* cells; a notion termed the ‘*S. aureus* head-start hypothesis’ owing to the earlier colonisation of *S. aureus* during childhood. When *P. aeruginosa* later colonises, it will inhabit an area outside of this inhibitory barrier and begin secreting *S. aureus*-inhibiting substances to maintain the distance from *S. aureus* cells^48^. This mutual antagonism allows co-existence of these species, which is one example of where the interaction could perhaps be described as both antagonistic and cooperative^48^. Figure 3 provides a diagrammatic representation of the inhibitory molecules associated within this mutually antagonistic interaction; however, it is important to consider the influence of other factors on the frequency of their production. For example, the degree of alginate produced by mucoid *P. aeruginosa* influences the release of *S. aureus*-inhibitory molecules (such as siderophores), with alginate overproduction contributing to their reduced release. The mucoid phenotype of *P. aeruginosa* is therefore thought to indirectly support co-colonisation with *S. aureus*, highlighting how this mutually antagonistic interaction can also be deemed cooperative^53^.

Strengthening the notion that *S. aureus* and *P. aeruginosa* are both antagonistic and cooperative is the idea that staphylococcal exoproducts (such as staphylopine (StP)) can modulate both types of interaction. StP was found to inhibit *P. aeruginosa* biofilm formation, thus outlining the competitive nature of this interaction. A cooperative angle was also found to be associated with staphylococcal exoproducts, though, with *P. aeruginosa* utilising the staphylococcal acetoin and citrate as carbon sources for its growth^54^. Furthermore, staphylococcal exoproduct *S. aureus* protein A has an anti-biofilm effect on *P. aeruginosa* clinical isolates, however the same molecule also prevents phagocytosis of *P. aeruginosa* by neutrophils^55^. This complexity emphasises the importance of interrogating the interspecies interactions within the CF lung, however the microbial diversity between each patient will compromise our ability to fully understand these intricacies clinically (Section “The CF lung environment facilitates the colonisation of diverse polymicrobial communities. ”).

Such patient-to-patient differences can be somewhat attributed to the duration of infection, with the inhibitory capacity of *P. aeruginosa* against *S. aureus* varying depending on the patient’s duration of infection^56^. The interactive ability of different species is also different depending on the bacterial load, with *Bacillus subtilis* effecting *P. aeruginosa* biofilms to varying degrees depending on inoculation density^57^. These additional variables demonstrate the diversity between each individual and ultimately emphasises the scale of what remains to be elucidated regarding the interspecies interplay in CF.

The interspecies interactions within CF extend far beyond what has been described between *S. aureus* and *P. aeruginosa*. Table 1 provides a more in-depth summary of the interspecies interactions taking place in the CF lung, for species aside from *P. aeruginosa* and *S. aureus*.

**Table 1:** Current perspectives regarding the interspecies interactions in the CF airway

Pathogen	Interacting species	Mechanisms of the interaction
*Burkholderia multivorans*	*Burkholderia cenocepacia*	**Competitive -** *B. multivorans* can secrete non-proteinaceous factor(s) that inhibit *B. cenocepacia*^98^.
	*Pandoraea apista*	**Competitive -** *B. multivorans* can secrete proteinaceous factor(s) that inhibit *P. apista*^98^.
	*Burkholderia thailandensis*	*B. thailandensis* uses CDI system proteins to control *B. multivorans* growth during co-culture^99^.
*Pseudomonas aeruginosa*	*Rothia mucilaginosa*	**Cooperative** - *P. aeruginosa* uses metabolites produced by *R. mucilaginosa* as precursors for the synthesis of its own primary metabolites, such as glutamate^100^.
	*Achromobacter xylosoxidans*	**Species dependent –** *A. xylosoxidans* can both facilitate and inhibit the growth, motility and pigmentation of *P. aeruginosa*, depending on the species tested^62^.
	*Aspergillus fumigatus*	**Two-step event** – When these two species are separated in the lungs, *P. aeruginosa* will release volatile compounds to support the invasion of *A. fumigatus*, thus suggesting **cooperation**. However, once they’re in direct contact, the interaction becomes **mutually anatagonistic**^101^. *P. aeruginosa* inhibits *A. fumigatus* through many mechanisms including phenazine secretion (such as pyocyanin). In addition, the *P. aeruginosa* siderophore pyoverdine can inhibit *A. fumigatus* biofilms. *A. fumigatus* is also anti-pseudomonal, with gliotoxin being the main agent for this. This **mutual antagonism** allows these species to co-exist following initial colonisation^42,102–104^.
	*Burkholderia cenocepacia – Burkholderia cepacia complex*	In a murine model, *B. cenocepacia* positively influenced *P. aeruginosa* biofilm development by increasing biofilm biomass^105^. This **cooperative** interaction also goes the opposite way, with alginate production by *P. aeruginosa* inhibiting the host immune response to promote *B. cenocepacia* persistence^106^. CF clinical isolates of both species have also been shown to produce bacteriocin-like toxins, suggesting that they would have inhibitory effects on other species^107^. This interaction is incredibly complex, with the *B. cenocepacia* bacteriocin Tailocin showing cidal activity against *P. aeruginosa* and thus also suggesting a degree of **antagonism** between these otherwise cooperative species^97^. There is also antagonistic activity of *P. aeruginosa* supernatants towards *B. cenocepacia*, that is dependent on an intact alkyl quinolone signalling system (within *P. aeruginosa*). Pyoverdine produced by *P. aeruginosa* is inhibitory of *B. cenocepacia*^98^, and rhamnolipids produced by *P. aeruginosa* can modulate the colony morphotype and swarming motility of *B. cenocepacia*^108^.
	*Burkholderia multivorans – Burkholderia cepacia complex*	*B. multivorans* can inhibit *P. aeruginosa*, however *P. aeruginosa* can also inhibit *B. multivorans*. This suggests a **competition** during co-infection, which will result in an overall decrease in both species^98^.
	*Stenotrophomonas maltophilia*	**Cooperative**^41,109^ **and competitive**^62^ – Infection with *P. aeruginosa* increases the persistence of *S. maltophilia*^41^ due to an increased expression of adherence and chemotaxis genes in *S. maltophilia* during co-infection. Greater adherence of *S. maltophilia* to CF bronchial epithelial cells was observed in *P. aeruginosa* pre-treated cells compared to untreated controls^109^. Similarly, *P. aeruginosa* has been shown to become more motile in the presence of *S. maltiphilia*^62^. However, a **competitive** angle is also seen, with *P. aeruginosa* presence reducing *S. maltophilia* growth^62^.
	*Achromobacter insuavis*	Exoproducts of *A. insuavis* prevent adhesion of *P. aeruginosa*, which is essential for its biofilm formation. As a result, *P. aeruginosa* aggregates within a biofilm are smaller in the presence of *A. insuavis*^110^, suggesting **competition**.
	Aninosus group streptococci – *S. anginosus, S. constellatus, S. intermedius*	Can exist in a biofilm co-culture suggesting **cooperation**. Co-colonisation (with CF2004 *P. aeruginosa* strain) enhances the production of the virulence factors elastase and pyocyanin, suggesting that multispecies colonisation can increase pathogenicity^111^.
	*Streptococcus sanguinis*	Co-culture with *P. aeruginosa* PAO1 can enhance the number of viable *S. sanguinis* SK36 cells in a biofilm by 100- to 1,055-fold, compared to monoculture. This was attributed to an increase in *S. sanguinis* growth when in the presence of *P. aeruginosa*. This **cooperative** interaction was one-sided, with *S. sanguinis* presence not affecting *P. aeruginosa* growth. *S. sanguinis* growth enhancement was especially true for mucoid (compared to non-mucoid) *P. aeruginosa* strains. However, *P. aeruginosa* mutants that overproduced siderophores failed to promote the growth of *S. sanguinis*, thus suggesting that iron competition is an important means of interaction for these two species^112^.
	*Candida albicans*	Complex interaction where *P. aeruginosa* virulence is both supported and attenuated^113^. Phenazine production by *P. aeruginosa* up-regulates *C. albicans* ethanol production^114^. Ethanol has been shown to stimulate biofilm formation in *P. aeruginosa* via activation of transcription from the alginate promoter *algD*^115^. Ethanol also acts as an immunosuppressant, preventing clearance of *P. aeruginosa* in the lungs by preventing macrophage recruitment in a rat model^116^. In this sense, the interaction is **cooperative**. Conversely, though, *P. aeruginosa* virulence is reduced by *C. albicans* since the yeast suppresses expression of the siderophores pyochelin and pyoverdine^117^ – thus also suggesting **competition**.
	*Pandoraea* species - *P. pulmonicola* and *P. apista*	Quantification of bacterial DNA within mixed cultures, using real-time PCR, indicates that *P. aeruginosa* has an inhibitory effect on these two *Pandoraea* species^98^, outlining **competition**.
	*Pseudomonas putida*	PQS released from *P. aeruginosa* has been shown to reduce swarming motility, reduce biofilm formation and interfere with iron uptake within *P. putida –* thus eliciting an **inhibitory** effect. As such, PQS has been termed a *P. aeruginosa* ‘chemical weapon’ against competitors^118^, also having an inhibitory effect on multiple Gram positive and Gram negative bacteria including *Bacillus cereus* and Stenotrophomonas species, respectively^119^.
	*Mycobacterium abscessus* complex	*M. abscessus* subsp. *abscessus* has a **competitive** advantage over *P. aeruginosa*. *P. aeruginosa* secretes HHQ and PQS, which elicit antimicrobial effects against other pathogens. *M. abscessus* subsp. *abscessus* (but not the remaining two subspecies) expresses *aqdRABC* genes, which contribute to degradation of HHQ and PQS to enable this subspecies to persist. When an *M. massiliense* isolate lacking *aqd* genes was co-cultured with *P. aeruginosa*, PQS production significantly increased – thus suggesting that *P. aeruginosa* attempts to respond to *M. abscessus* infection through this mechanism. This interplay is therefore subspecies dependent^120^. A **competitive** advantage of *M. abscessus* over *P. aeruginosa* has also been demonstrated in dual-species biofilms of these species during antibiotic treatment^66,67^.
*Stentrophomonas maltophilia*	*Achromobacter xylosoxidans*	**Competitive** – *S. maltophilia* typically shows reduced growth and motility in the presence of *A. xylosoxidans*^62^.
	*Staphylococcus aureus*	**Competitive** – *S. maltophilia* shows reduced motility in the presence of *S. aureus*^62^.

*CDI* contact-dependent growth inhibition, *DNA* deoxyribose nucleic acid, *PCR* polymerase chain reaction, *PQS* 2-heptyl-3-hydroxy-4(1H) quinolone, *HHQ* 2-heptyl-4(1H)-quinolone.

### Intraspecies interactions within CF polymicrobial communities

Adding further complexity is the potential for intraspecies interactions, whereby, unlike those in Section “Interspecies interactions within CF polymicrobial communities.”, microorganisms of the same species can interact with one another. It is well known that genomically diverse isolates of the same species can be associated with CF, being evidenced for both *S. aureus*^58^ and *P. aeruginosa*^59,60^, thus raising opportunity for intraspecies interactions—although these are generally less studied compared to interspecies alternatives. Thus, little is known about how pathogens of the same species may influence one another, only gaining attention in more recent years. Of interest is the potential for secreted molecules to impact different strains of the same species; for example, R type pyocin-producing strains of *P. aeruginosa* appear to predominate over non-producers, perhaps due to the pyocins exerting an antimicrobial effect over the non-producing strains^61^. Similar work has demonstrated how co-culture of different *Achromobacter xylosoxidans* strains result in an overall increase in bacterial motility; a finding that was also observed for *P. aeruginosa*^62^. This work highlights the complexity of the interactions taking place CF microbial populations, raising a requirement for further studies into the intraspecies interactions occurring in vivo.

### The relationship between polymicrobial communities and antimicrobials

Polymicrobial communities are thought to lead to the development of microbial ecosystems within the lung, whereby microbial interactions can influence a therapeutic outcome. It is therefore important to consider these populations during antimicrobial discovery, to enable better transferability of outcomes from the laboratory to the clinic.

The relationship between bacterial communities and antimicrobials takes two angles, where either: (1) the antimicrobials influence the polymicrobial community, or (2) the bacterial community impacts antibiotic activity^63^. There is therefore an extremely complex interplay between these two factors, with them impacting each other in tandem to result in diverse treatment outcomes. The unique microbial composition of each patient is therefore likely to influence antimicrobial activity, perhaps contributing to the persistence of infection despite antibiotic therapy. One example of the former angle relates to *P. aeruginosa* and *Streptococcus constellatus* colonisation*;* tobramycin has been shown to perpetuate antibiotic tolerance in a mixed species biofilm model of these species, with the addition of tobramycin enhancing *S. constellatus* biofilm formation. This was linked to a tobramycin-induced reduction in *P. aeruginosa* rhamnolipid production, which would otherwise reduce *S. constellatus* biofilm viability in the absence of tobramycin^64^. Considering that tobramycin is a frontline maintenance antibiotic used in CF infections^49^, this observation is extremely important and highlights how antimicrobial use may perpetuate the persistence of polymicrobial CF infections in vivo.

The reverse can also occur, whereby the bacterial community can affect antibiotic susceptibility. This is similarly important for tobramycin, with *P. aeruginosa* becoming sensitised to this antibiotic in a mixed biofilm community of *S. aureus, Streptococcus sanguinis* and *Prevotella melaninogenica* versus monoculture^65^. Similarly, the response of *M. abscessus* biofilms to antibiotics is influenced by the presence of *P. aeruginosa*; in a dual-species biofilm of these species, ceftazidime showed more effective biofilm inhibition than the single-species alternative of *M. abscessus* alone^66,67^. Another example includes the upregulation of the *S. aureus tet38*, *norA* and *norC* genes in the presence of *P. aeruginosa*, which encode antibiotic pumps and thus confer increased tolerance to tetracycline and ciprofloxacin^68^.

**Fig. 4:**
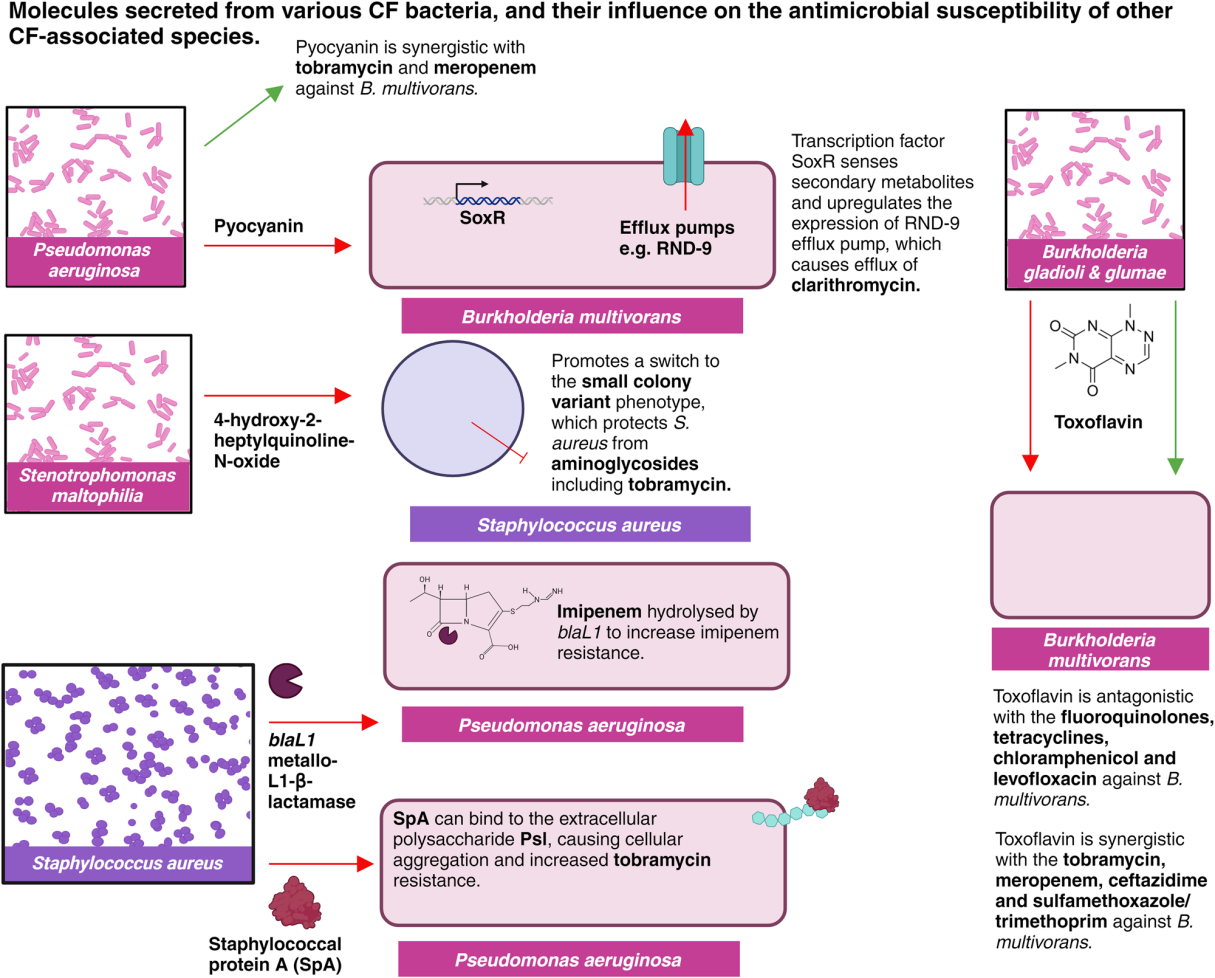
Molecules secreted from various CF bacteria, and their influence on the antibiotic susceptibility of co-colonising species^5,63,69,137,138^. Figure created on Biorender.com, accessed 21 August 2024. RND-9, resistance nodulation division 9.

In addition to these interactions, bacterial pathogens can secrete secondary metabolites within the lung, such as the pyocyanin secreted from *P. aeruginosa* and the toxoflavin produced by *Burkholderia gladioli*^69^. These secondary metabolites have the potential to influence treatment outcomes, by inducing efflux systems, modulating oxidative stress responses or effecting redox homeostasis^70^. Thus, it is important to review the impact of various secondary metabolites on antimicrobial susceptibility, to gauge a better understanding of the influence of polymicrobial communities on treatment success. Considering this complexity during antibiotic screening and discovery may provide significant promise to improve the predictability of drug susceptibility assays, with the addition of pyocyanin and toxoflavin to *B. multivorans* drug screening increasing the MICs of multiple tetracyclines, fluoroquinolones and chloramphenicol^69^. Such influence of secondary metabolites may, therefore, be a driving force for the poor clinical success associated with antibiotic treatment; thus, raising the question of how we can tailor treatment approaches to consider these diverse microbial ecosystems. Identification and quantification of 2-heptyl-4(1H)-quinolone, 2-heptyl-3-hydroxy-4(1H)-quinolone and pyocyanin from *P. aeruginosa* has been achieved in cultures derived from CF sputum samples, for example, using dispersive liquid-liquid microextraction followed by matrix-assisted laser desorption/ionization mass spectrometry. This advancement raises promise for the use of such systems in clinical settings, enabling a greater understanding of each patient’s unique microbial population and thus helping to inform more efficacious and personalised treatment^71^. Figure 4 provides a summary of the various molecules secreted from different CF bacteria, and their effects on antimicrobial susceptibility in different species.

## Discussion

Unravelling the complex polymicrobial communities within CF is a major challenge, with the multifaceted nature of the topic posing endless research potential. However, such understanding raises the opportunity to massively improve antimicrobial discovery, with much research highlighting how influential these intricate bacterial communities are on compound susceptibility (Section “The relationship between polymicrobial communities and antimicrobials. ”). Consideration of polymicrobial interactions during drug discovery could offer significant promise to improve physiological relevance, and thus enhance the translation of outcomes from the laboratory to the clinic. However, standardisation of one universal assay is going to be challenging, with the microbial diversity between patients (Section “Polymicrobial infection in cystic fibrosis”) warranting a more personalised approach. We will discuss these challenges in this section, as well appraising the accuracy of the currently used antimicrobial sensitivity assays.

### Antimicrobial screening and discovery is typically planktonic and monomicrobial

The standardised guidelines for antimicrobial screening and discovery involve broth microdilution, whereby serial dilutions of a compound are incubated in the presence of a planktonic monoculture in microtiter plates. This setup is monitored using either colony counting^72^ or optical density readings^73^, to ascertain how the species responds to different concentrations of compound over time. Resazurin is typically added to each well at endpoint, acting as a viability indicator to determine the minimum antibiotic concentration required to cause bacterial inhibition (minimum inhibitory concentration, MIC). In the presence of viable bacterial cells, reduction of resazurin to resorufin is indicated by a purple-to-pink colour change—thus, the lowest antibiotic concentration to show a purple coloration is identified as the MIC^74^. The European Committee on Antimicrobial Susceptibility Testing provides a series of MIC breakpoints, indicative of whether a species is resistant or sensitive to a particular antibiotic following this methodology (https://www.eucast.org/clinical_breakpoints).

Although this process provides a relatively simple indication of how a species may respond to a compound, the lack of physiological relevance associated with this technique poses major challenges when compounds are translated in vivo. As such, the MIC identified in this basic setup is not predictive of the concentrations required in vivo^20^—especially considering that these assays are typically monomicrobial. Polymicrobial interactions have been shown to have a profound impact on antimicrobial susceptibility, thus raising a demand to develop more sophisticated polymicrobial drug screening models that consider these more resistant phenotypes (Section “The relationship between polymicrobial communities and antimicrobials”)^66,67,75^. It is undoubtedly a challenge to estimate how a standardised MIC assay will predict treatment success or failure patients on the whole, since each patient’s response to an antibiotic will differ depending on many variable parameters^76^. However, it is reasonable to suggest that small improvements in physiological relevance, such as those proposed here, will bring these assays one step closer to better representing clinical outcomes.

### Challenges to the development of polymicrobial *M. abscesses* drug screening models

Despite this need to develop polymicrobial drug screening assays, there are major challenges that will compromise the ability to develop one standardised approach. One overwhelming obstacle is the widespread diversity seen between each patient, as described in Section “Polymicrobial infection in cystic fibrosis”. This diversity means that no model will be perfectly representative of each unique case, and so it is perhaps unreasonable to imagine an in vitro assay that is predicative of all in vivo outcomes. Furthermore, each patient is likely to be infected with different MABSC strain(s), and each strain will undoubtedly respond to antibiotics differently. This limitation raises the need for a more personalised approach, whereby each patient is assessed in extensive detail before informing treatment plans; a requirement that so far remains unachievable within medicine. Recent advancements in the field of artificial intelligence raise promise for its application to antimicrobial susceptibility testing^77^, however, these systems raise inequity challenges as they’re generally only achievable within wealthy countries. Although incredibly promising, the development of such a system would be costly and would perhaps not be attainable on a global scale^78^.

Another obstacle surrounds the variable growth rates and competition profiles of each species, which is especially important for NTMs that tend to grow slowly compared to other non-mycobacterial bacteria. To determine the efficacy of a compound, bacterial cells must be monitored before and after treatment to ascertain viability—a process that is achieved in monocultures using basic colony counting^72^ or optical density readings^73^ (Section “Antimicrobial screening and discovery is typically planktonic and monomicrobial”). However, the variable growth rates of each species will raise challenges regarding the standardisation of bacterial number throughout a polymicrobial assay, meaning that controlled enumeration of cells following treatment may become problematic. It is possible to control the inoculum introduced at the start of an experiment (such as via the use of a standardised number of colony forming units per millimetre (CFU/mL))^67^, however the different growth rates and competition profiles of each species will compromise the ability to compare the CFU/mL output following treatment. To overcome this, continuous flow models have been developed to enable maintenance of cell number in polymicrobial communities over time, such as between *S. aureus*, *C. albicans* and *P. aeruginosa*. These systems can allow for the maintenance stable communities despite each species outcompeting on another in batch culture^79^, and are robust and considerably cheaper than existing in vivo infection models. This level of sophistication, however, is yet to be applied to *M. abscessus*. Application of similar systems to *M. abscessus* drug screening could allow greater understanding of how antimicrobials behave in a polymicrobial context, as typically exhibited in vivo.

### Factors to consider in the development of polymicrobial infection models

Despite being incredibly important when considering physiological relevance, polymicrobial infection isn’t the only factor that must be studied in the development of translational drug discovery models. The CF lung environment extends far beyond this, with another influential host factor including anaerobiosis—induced by the thick mucus plugging in the CF^80^. Outlining the importance of this is the interplay between *S. aureus* and *P. aeruginosa*, with the hypoxic lung environment being thought to facilitate cohabitation of these two species in both mixed species biofilms and planktonic co-culture^81^. Since drug screening assays typically employ aerobic cultures, the influence of hypoxia is underrepresented and may contribute to inaccuracies during in vitro screening and drug discovery. The impact of hypoxia must therefore inform future studies, adding yet another variable to consider in the development of polymicrobial infection models.

It is also important to consider host cells and bacterial physiology during the development of translational drug screening models. Within the lung, *M. abscessus* has been shown to adopt a biofilm phenotype, with extracellular matrix-surrounded *M. abscessus* microcolonies being detected in the intra-alveolar walls of a patient with CF^82^. Biofilms have been shown to increase the inhibitory concentration of antibiotics by 100-1000x in some species^83^, and thus a failure to consider this phenotype in vitro would falsely predict antimicrobial tolerance within the body. Furthermore, lung epithelial cells are thought to influence a bacterium’s response to antibiotics, adding an additional layer of complexity. When culturing a dual-species biofilm of *M. abscessus* and *P. aeruginosa* on the human epithelial cell line A549, the activity of clarithromycin decreased; likely since this antibiotic can accumulate intracellularly and therefore reduce clarithromycin bioavailability^66^. It is therefore vital to consider the impact of both host cells and biofilm phenotypes in the development of a translational, polymicrobial drug screening model.

Furthermore, many studies have shown how media choice affects microbial interactions, further highlighting the need to recapitulate the lung environment^84–86^. Since 1997, two lineages of sputum mimetic media have been developed, namely artificial sputum media^87,88^ and synthetic cystic fibrosis sputum media^89^—with many compositional adaptations taking place to each recipe over the last few decades^90–95^. Both media types are formulated based on the average concentrations of different nutrients found within sputum, thus offering potential to better represent the metabolism of host infection. An array of bacterial species can grow in this optimised media^90,94,96^, albeit slightly slower compared to the standard media 7H9 in the case of *M. abscessus* in artificial CF sputum^94^. This provides opportunity for researchers to apply such media types to drug discovery, beneficial since this media is based on the average concentrations of sputum metabolites found within a cohort of CF subjects. As such, these media types are theoretically representative of the CF population on average, and therefore they can provide better representation of the clinical situation^89^. Aside from more recent work^65^, polymicrobial assays typically make use of standard laboratory media such as tryptone nutrient broth^97^, and thus we cannot fully estimate the relevance of their results. Future CF polymicrobial studies should consider the application of sputum mimetic media to better represent such communities in vivo, and thus help better predict clinical outcomes.

## Conclusions and future directions

Although much is known about interspecies interactions within CF, most of this research surrounds the ‘core’ CF pathogen *P. aeruginosa*. By considering the interplay between antimicrobials and polymicrobial communities in conjunction with our need for improved *M. abscessus* treatment, we imply a requirement for further research into the polymicrobial nature of *M. abscessus*. We demonstrate how application of polymicrobial communities to *M. abscessus* drug screening offers significant promise to improve the predictability of in vitro assays. Consideration of physiologically relevant media and conditions (such as sputum media, hypoxia, and host cells) would optimise the accuracy of polymicrobial assays even further, by helping raise a phenotype closer to that exhibited in vivo. This change in approach is likely to inform more efficacious antibiotic regimens; a strategy that is beneficial for both antibiotic stewardship and patient outcome. The success of future antibiotic discovery for *M. abscessus* infection is dependent on the consideration of factors here discussed.
